# Hepatocyte growth factor ameliorates dermal sclerosis in the tight-skin mouse model of scleroderma

**DOI:** 10.1186/ar2068

**Published:** 2006-10-18

**Authors:** Tsuyoshi Iwasaki, Takehito Imado, Sachie Kitano, Hajime Sano

**Affiliations:** 1 Division of Rheumatology and Clinical Immunology, Department of Internal Medicine, Hyogo College of Medicine, 1-1 Mukogawa-cho, Nishinomiya, Hyogo 663-8501, Japan; 2 Division of Hematology, Department of Internal Medicine, Hyogo College of Medicine, 1-1 Mukogawa-cho, Nishinomiya, Hyogo 663-8501, Japan

## Abstract

The tight-skin (TSK/+) mouse, a genetic model of systemic sclerosis (SSc), develops cutaneous fibrosis and defects in pulmonary architecture. Because hepatocyte growth factor (HGF) is an important mitogen and morphogen that contributes to the repair process after tissue injury, we investigated the role of HGF in cutaneous fibrosis and pulmonary architecture defects in SSc using TSK/+ mice. TSK/+ mice were injected in the gluteal muscle with either hemagglutinating virus of Japan (HVJ) liposomes containing 8 μg of a human HGF expression vector (HGF-HVJ liposomes) or a mock vector (untreated control). Gene transfer was repeated once weekly for 8 weeks. The effects of *HGF *gene transfection on the histopathology and expression of tumor growth factor (TGF)-β and IL-4 mRNA in TSK/+ mice were examined. The effect of recombinant HGF on IL-4 production by TSK/+ CD4^+ ^T cells stimulated by allogeneic dendritic cells (DCs) *in vitro *was also examined. Histologic analysis revealed that *HGF *gene transfection in TSK/+ mice resulted in a marked reduction of hypodermal thickness, including the subcutaneous connective tissue layer. The hypodermal thickness of HGF-treated TSK/+ mice was decreased two-fold to three-fold compared with untreated TSK/+ mice. However, TSK/+ associated defects in pulmonary architecture were unaffected by *HGF *gene transfection. *HGF *gene transfection significantly inhibited the expression of IL-4 and TGF-β1 mRNA in the spleen and skin but not in the lung. We also performed a mixed lymphocyte culture and examined the effect of recombinant HGF on the generation of IL-4. Recombinant HGF significantly inhibited IL-4 production in TSK/+ CD4^+ ^T cells stimulated by allogeneic DCs. *HGF *gene transfection inhibited IL-4 and TGF-β mRNA expression, which has been postulated to have a major role in fibrinogenesis and reduced hypodermal thickness, including the subcutaneous connective tissue layer of TSK/+ mice. HGF might represent a novel strategy for the treatment of SSc.

## Introduction

Systemic sclerosis (SSc) is a connective tissue disorder of unknown etiology that is characterized by an excessive deposition of extracellular matrix protein in the affected skin, in addition to various internal organs. Currently, no effective therapies for SSc exist [1]. The tight-skin (TSK/+) mouse is a genetic model for human SSc. Although the phenotypic characteristics of the TSK/+ mouse are not identical to those of human SSc patients, TSK/+ mice produce autoantibodies against SSc-specific autoantigens, including topo-I, fibrillin 1 (fbn-1), collagen type 1, and Fcγ receptors [2,3]. The gene defect responsible for the TSK/+ phenotype in mice is yet to be definitively identified; however, the *fbn-1 *gene has been recently proposed as a candidate locus for this disorder [4]. The *fbn-1 *gene mutation seems to provide an explanation for the embryonic lethality of homozygous tight skin (TSK/TSK) animals. Heterozygous (TSK/+) mice, which have a normal lifespan, exhibit fibrosis and thickening of subcutaneous dermal tissue. Other abnormalities in TSK/+ mice include increased lung collagen content, enlarged air spaces reminiscent of pulmonary emphysema, and, with advanced age, development of progressive myocardial fibrosis and hypertrophy [5-7].

Hepatocyte growth factor (HGF) was originally identified and cloned as a potent mitogen for hepatocytes [8,9]. It has mitogenic, motogenic, and morphogenic effects on various epithelial tissues, including the liver, kidneys, lungs, and intestines [10-12]. HGF also shows antiapoptotic activity [13] and has a role in suppressing fibrosis in the liver [14]. HGF might, therefore, induce tissue repair in dermal sclerosis associated with SSc. We recently demonstrated that repeated transfection of the human *HGF *gene into skeletal muscle induced continuous production of HGF, strongly inhibited acute graft-versus-host disease (GVHD) after allogeneic hematopoietic stem-cell transplantation (HSCT), and protected against thymic damage caused by GVHD in a well-characterized mouse model of GVHD [15,16]. The present study was performed to examine the therapeutic effect of HGF on tissue fibrosis in TSK/+ mice.

## Materials and methods

### Animals

TSK/+ and homozygous pallid (pa/pa) mutant mice with a C57BL/6 background were obtained from the Jackson Laboratory (Bar Harbor, ME, USA). TSK/+ mice are heterozygous for both *TSK *and *pa *gene mutations. TSK/+ mice were produced by mating TSK/+ male mice with pa/pa female mice. TSK/+ progeny were identified by their back-coat and eye colors, in addition to their characteristic loss of skin pliability. To verify the TSK/+ genotype, PCR amplification of a partially duplicated *fbn-1 *gene was carried out using genomic DNA from each mouse, as described previously [17]. C57BL/6 and (C57BL/6 × DBA/2)F1 (BDF1) mice were obtained from the Shizuoka Laboratory Animal Center (Hamamatsu, Shizuoka, Japan). All mice were maintained in a pathogen-free facility at the Hyogo College of Medicine (Nishinomiya, Hyogo, Japan). Animal experiments were performed in accordance with the guidelines of the National Institutes of Health (Bethesda, MD, USA), as specified by the animal care policy of Hyogo College of Medicine.

### Expression vector and preparation of liposomes containing hemagglutinating virus of Japan (HVJ)

Human HGF cDNA (2.2 kb) was inserted into the EcoRI and NotI sites of the pUC-SRα plasmid under control of the cytomegalovirus enhancer-promoter [18]. HVJ liposomes containing plasmid DNA and high mobility group 1 protein were constructed, as described previously [15,16]. Briefly, phosphatidylserine, phosphatidylcholine, and cholesterol were mixed at a weight ratio of 1 : 4.8 : 2. This lipid mixture (1 mg) plus plasmid DNA (20–40 μg), which had previously been complexed with 6–12 μg of high mobility group 1 nonhistone chromosomal protein purified from calf thymus, were sonicated to form liposomes and then mixed with ultraviolet-irradiated HVJ. Excess free virus was subsequently removed from the HVJ liposomes by sucrose-density-gradient centrifugation.

### Gene transfer

TSK/+ mice were injected in the gluteal muscle with either HVJ liposomes containing 8 μg of the human HGF expression vector (HGF-HVJ liposomes) or mock vector (untreated control). Gene transfer was repeated once weekly for 8 weeks.

### Histopathology

Tissues were fixed in 10% buffered formalin and embedded in paraffin. Sections were stained with hematoxylin and eosin and were examined by light microscopy.

### RT-PCR

RNA was extracted using an Isogen (Nippongene, Toyama, Japan) kit, according to the manufacturer's instructions, and cDNA was prepared using 2.5 μM random hexamers (Applied Biosystems Inc., Foster City, CA, USA). IL-4 and TGF-β mRNA levels were quantified by real-time RT-PCR in a total volume of 25 μl for 40–50 cycles of 15 seconds at 95°C and 40–50 cycles of 1 minute at 60°C. Samples were run in triplicate and relative expression levels were determined by normalizing expression levels to that of β-actin. The primer sequences used were as follows:

1. IL-4, CCAGCTAGTTGTCATCCTGCTCTTCTTTCTCG and CAGTGATGAGGACTTGGACTCATTCATGGTGC.

2. TGF-β1, TGGACCGCAACAACGCCATCTATGAGAAAACC and TGGAGCTGAAGCAATAGTTGGTATCCAGGGCT.

3. β-actin, TGTGATGGTGGGAATGGGTCAG and TTTGATGTCACGCACGATTTCC.

### Mixed lymphocyte reaction (MLR) and in-vitro cytokine production

CD4^+ ^T cells and CD11c^+ ^dendritic cells (DCs) were purified from spleen cells using immunomagnetic beads (Miltenyi Biotec, CA, USA). The purity of the CD4^+ ^and CD11c^+ ^cell populations was >90% and >95%, respectively. CD4^+ ^T cells from TSK/+ (H-2^b^) mice (4 × 10^6 ^cells/ml/well) were cultured with irradiated (20 Gy) CD11c^+ ^DCs from BDF1 (H-2^b × d^) mice (1 × 10^6 ^cells/ml/well) in 24-well flat-bottomed plates (Falcon Labware, Lincoln Park, NJ, USA). After 72 hours, viable cells (1 × 10^5 ^cells/200 μl/well) were stimulated in 96-well flat-bottomed plates (Falcon Labware) coated with 5 μg/ml anti-CD3 mAb. After 48 hours, IL-4 and IFN-γ concentrations in the culture supernatants were measured by ELISA.

### ELISA for IL-4

The levels of murine IL-4 in culture supernatants were measured by ELISA using antimouse IL-4 mAb (Genzyme Pharmaceuticals, Cambridge, MA, USA) according to the manufacturer's protocol.

### Statistical analysis

Group mean values were compared by the two-tailed Student's *t*-test. A *p *value of < 0.05 was considered statistically significant.

## Results

### Prevention of scleroderma by *HGF *gene transfection

**Figure 1:**
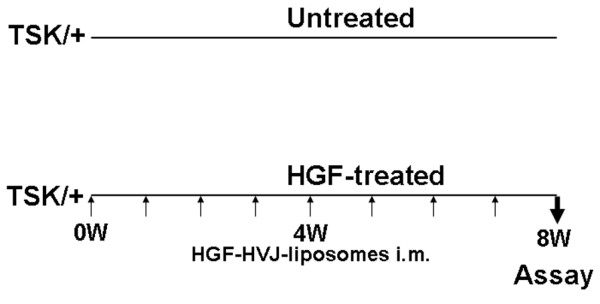
Experimental protocol for injection of HGF-HVJ liposomes into TSK/+ mice. Treatment consisted of once-weekly injection of either HGF-HVJ liposomes or control mock vector for a period of 8 weeks. Treatment was initiated at the age of 4 weeks. Histopathology and cytokine mRNA expression in the spleen, skin, and lungs was examined 8 weeks after treatment. HGF, hepatocyte growth factor; HVJ, hemagglutinating virus of Japan; im, intramuscular; TSK/+, tight skin.

**Figure 2:**
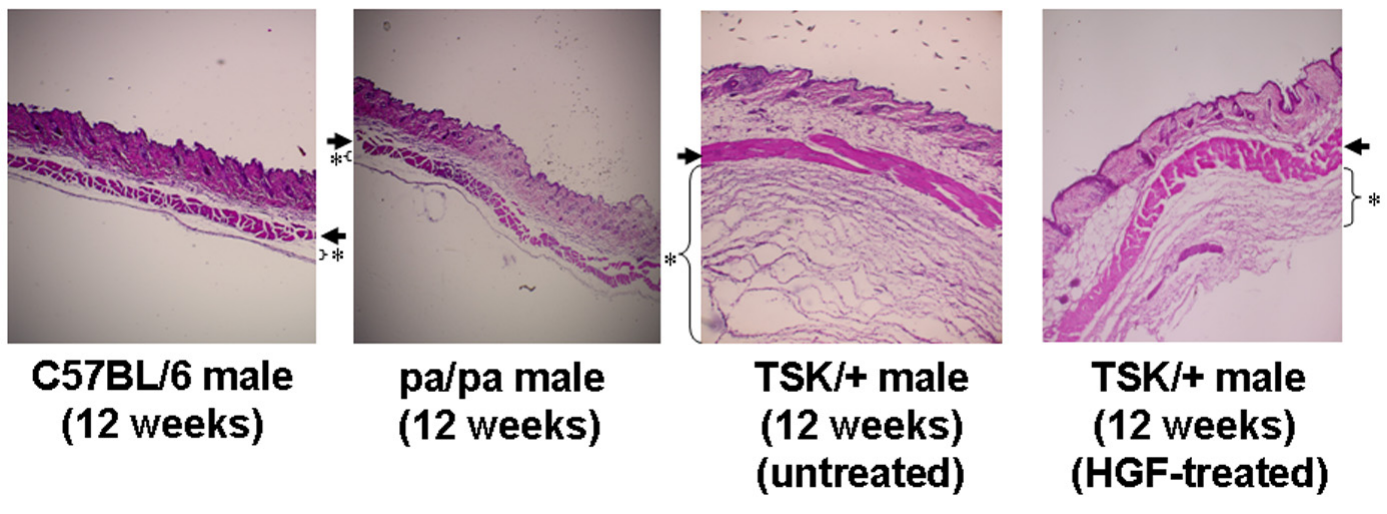
Effect of HGF on skin fibrosis in TSK/+ mice. Skin fibrosis in dorsal skin from C57BL/6, pa/pa, and TSK/+ mice with or without treatment with HGF. Representative histologic sections stained with hematoxylin and eosin are shown (× 40). An asterisk indicates the subcutaneous loose connective tissue layer beneath the panniculus carnosus (arrow). HGF, hepatocyte growth factor; TSK/+, tight skin.

**Figure 3:**
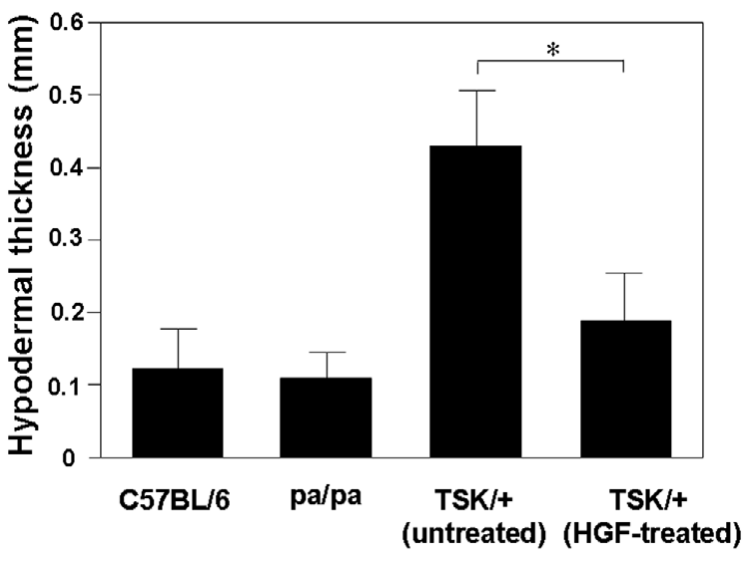
Effect of HGF on skin fibrosis in TSK/+ mice. Skin fibrosis was assessed by quantitatively measuring hypodermal thickness. The hypodermal thickness was measured under light microscopy as the thickness of the hypodermis or superficial fascia beneath the panniculus carnosus. Data represent the mean ± standard deviation from six mice (three male mice and three female mice) in each group. *p < 0.05. HGF, hepatocyte growth factor; TSK/+, tight skin.

We previously reported that repeated transfection of the human *HGF *gene into skeletal muscle of allogeneic HSCT recipients reduced the tissue damage and subsequent inflammatory responses caused by acute GVHD [15,16]. To investigate the possible therapeutic effects of HGF on SSc, young TSK/+ mice were treated with *HGF *gene transfection. Treatment consisted of once-weekly intramuscular injection of either HGF-HVJ liposomes or control mock vectors for a period of 8 weeks (Figure 1). Histologic analysis revealed that HGF treatment of TSK/+ mice resulted in a marked reduction of hypodermal thickness, including the subcutaneous connective tissue layer (Figure 2). Skin fibrosis was assessed quantitatively by measuring hypodermal thickness. The hypodermal thickness of HGF-treated TSK/+ mice was decreased two-fold to three-fold compared with untreated TSK/+ mice (Figure 3).

### Effect of *HGF *gene transfection on pulmonary changes

**Figure 4:**
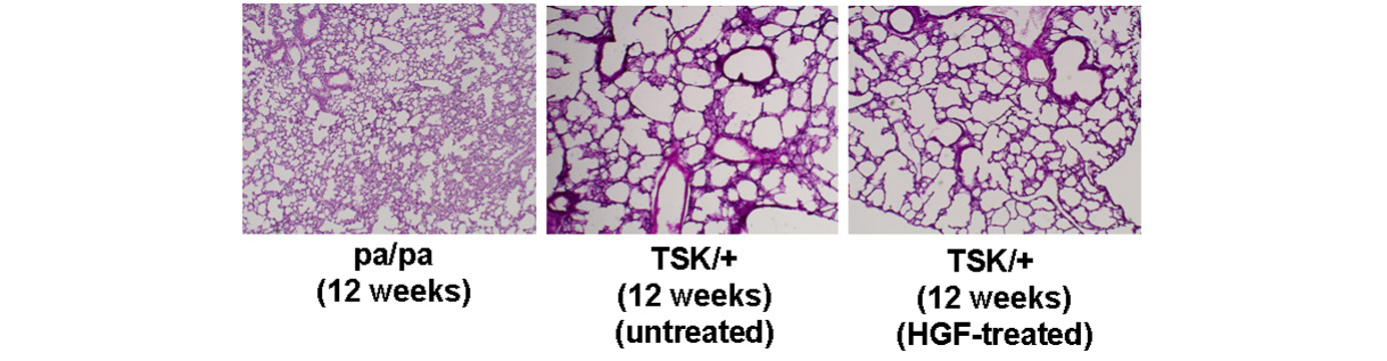
Effect of HGF on pulmonary changes in TSK/+ mice. Representative histologic sections stained with hematoxylin and eosin are shown (× 40). HGF, hepatocyte growth factor; TSK/+, tight skin.

The effect of *HGF *gene transfection on pulmonary changes was examined. Control mock-vector-transfected TSK/+ mice all displayed a markedly dilated alveolar space, with fewer alveolar walls compared with pa/pa mice. This TSK/+-associated abnormality in lung architecture was unaffected by *HGF *gene transfection (Figure 4).

### Effect of HGF on the expression of IL-4 and TGF-β mRNA expression and production

**Figure 5:**
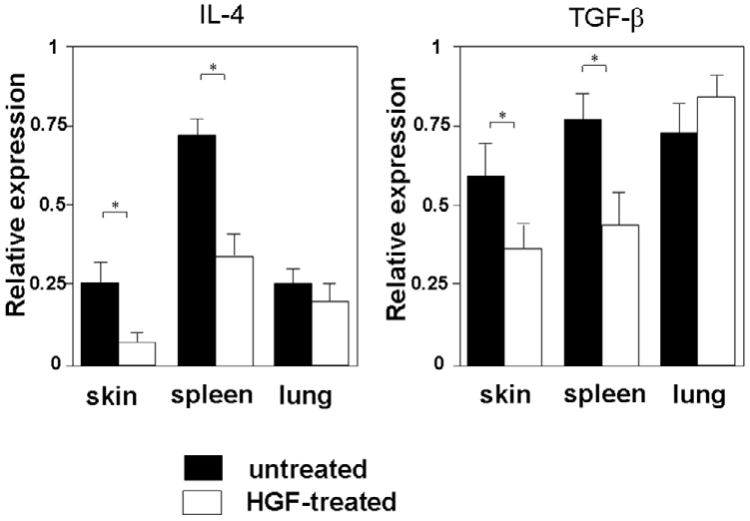
Effect of HGF on IL-4 and TGF-β1 mRNA expression in tight-skin mice. Total RNA was isolated from the skin, spleen, and lung, and IL-4 and TGF-β1 mRNA expression was analyzed by RT-PCR assay. Data represent the mean ± standard deviation from five mice. *p < 0.05. HGF, hepatocyte growth factor; IL, interleukin; TGF, tumor growth factor.

**Figure 6:**
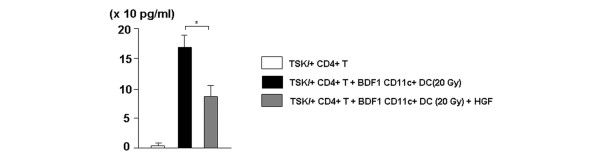
*In vitro* effect of HGF on IL-4 production by TSK/+ CD4^+ ^T cells. CD4^+ ^T cells from TSK/+ (H-2^b^) mice at the age of 6 weeks (4 × 10^6 ^cells/ml/well) were cultured with irradiated (20 Gy) CD11c^+ ^DCs from BDF1 (H-2^b × d^) mice (1 × 10^6 ^cells/ml/well) in the presence or absence of human recombinant HGF (10 ng/ml). CD4^+ ^T cells from TSK/+ (H-2^b^) mice (4 × 10^6 ^cells/ml/well) cultured without CD11c^+ ^DC stimulation were used as controls. After 72 hours, viable cells (1 × 10^5 ^cells/200 μl/well) were harvested and stimulated with anti-CD3 mAb (5 μg/ml) for 48 hours. The concentration of IL-4 in the culture supernatant was measured by ELISA. Data represent the mean ± standard deviation of three independent experiments. *p < 0.05. DC, dendritic cell; HGF, hepatocyte growth factor; T, T cell; TSK/+, tight skin.

IL-4 and TGF-β have been postulated to have major roles in fibrinogenesis in animal models [19-23]. To clarify whether modulation of IL-4 and TGF-β has a role in the prevention of sclerosis induced by *HGF *gene transfection in the scleroderma model mouse, we examined the mRNA expression of these cytokines. *HGF *gene transfection significantly inhibited the expression of IL-4 and TGF-β1 mRNA in the spleen and skin but not in the lung (Figure 5). We also performed MLR and examined the effect of HGF on the production of IL-4. Responder CD4^+ ^T cells from TSK/+ mice were cultured with irradiated (20 Gy) CD11c^+ ^DCs from BDF1 mice with or without recombinant HGF. After 3 days' culture, viable cells were stimulated by culture with anti-CD3 mAb for 48 hours and the IL-4 level in the culture supernatant was assayed by ELISA. HGF significantly inhibited IL-4 production from TSK/+ CD4^+ ^T cells stimulated by BDF1 DCs (Figure 6).

## Discussion

SSc is an autoimmune connective tissue disease that is characterized by microvascular damage, extracellular matrix deposition, and fibrosis. There is no completely effective treatment for this disease at present. We previously demonstrated that serum HGF levels were significantly elevated in patients with SSc and serum HGF levels correlated to markers of endothelial injury (thrombomodulin) and interstitial lung injury (KL-6), suggesting that elevation of serum HGF counteracts the endothelial and interstitial lung injury caused by SSc [24]. The serum level of HGF is significantly elevated in various diseases, depending on the severity of the disease [25-27]. However, endogenously induced HGF is not sufficient to repair tissue injuries, and, therefore, supplementation with exogenous HGF is necessary to accelerate the tissue repair process in animal models [14,15,28]. In the present study, we assessed the effect of exogenous HGF on skin fibrosis and the development of pulmonary defects in the TSK/+ mouse model of SSc. Both our present study and other previous studies [5] have shown that dermal thickness is similar in TSK/+ and wild-type littermates, but hypodermal thickness, including the subcutaneous connective tissue layer, is significantly increased in TSK/+ mice compared with wild-type littermates. *HGF *gene transfection of TSK/+ mice for a period of 8 weeks resulted in a marked reduction of hypodermal thickness, including the subcutaneous connective tissue layer. Although the therapeutic effect of HGF is not significant, we also observed the reduction of hypodermal thickness in TSK/+ mice following *HGF *gene transfection for a period of 4 weeks (data not shown).

Although the cause of SSc is unknown, IL-4 and TGF-β have been postulated to have major roles in fibrinogenesis. In one study, intravenously administered human immunoglobulin decreased splenocyte secretion of IL-4 and TGF-β, which resulted in abrogation of fibrinogenesis in TSK/+ mice and, consequently, prevented the accumulation of fibrous tissue [19]. Furthermore, administration of an anti-IL-4 or anti-TGF-β antibody prevented dermal collagen deposition in TSK/+ mice and murine sclerodermatous GVHD, respectively [20,21]. In the present study, HGF treatment also reduced expression of both IL-4 and TGF-β mRNA in the spleen and skin.

IL-4 regulates collagen and extracellular matrix production by fibroblasts [22,23]. TSK/+ mice exhibiting disrupted genes encoding IL-4 receptor alpha (IL-4Rα) or IL-4 lacked skin sclerosis [17,29], suggesting that IL-4 has a crucial role in skin sclerosis in TSK/+ mice. A primary source of IL-4 *in vivo *is CD4^+ ^T cells [30] and a previous study demonstrated that CD4^+ ^T cells were essential to the TSK/+ phenotype, because a lack of these cells prevented development of dermal thickening [31]. Therefore, we examined the effect of HGF on the generation of IL-4 from CD4^+ ^T cells. HGF significantly inhibited IL-4 production from CD4^+ ^T cells stimulated by allogeneic DCs, suggesting that HGF inhibits dermal fibrosis, in part, by inhibiting IL-4 production by CD4^+ ^T cells.

We also observed downregulation of TGF-β1 mRNA expression in TSK/+ mice by *HGF *gene transfection. TGF-β1 has a role in the induction of fibrosis, and *HGF *gene transfection inhibited the production of TGF-β1 from macrophage-like cells and fibroblastic cells [32]. Downregulation of TGF-β1 expression and inhibition of fibrosis by HGF were noted in a rat model of liver cirrhosis [14] and a mouse model of chronic renal failure [33]. Recently, HGF has been shown to downregulate TGF-β1 expression and prevent dermal sclerosis in a murine bleomycin-induced scleroderma model [34]. The authors observed that *HGF *gene transfection significantly reduced both the expression of TGF-β1 mRNA and the production of TGF-β1 by fibroblastic cells and macrophage-like cells that infiltrated the dermis. Furthermore, *HGF *gene transfection prevented the symptoms of not only dermal sclerosis, but also lung fibrosis induced by bleomycin injection.

By contrast, *HGF *gene transfection failed to alter the development of pulmonary abnormalities in TSK/+ mice in our study. The pathologic alteration of the lung structure of TSK/+ mice represents pulmonary emphysema and is not related to hypersynthesis of collagen that is similar to the pulmonary fibrosis associated with SSc [35]. Apparently, emphysema in TSK/+ mice is not owing to the mutated *fbn-1 *gene that is responsible for the occurrence of cutaneous hyperplasia, because transgenic mice bearing a mutated *fbn-1 *gene developed cutaneous hyperplasia but did not exhibit pulmonary emphysema [36]. Furthermore, TSK/+ mice exhibiting disrupted genes encoding IL-4Rα, TGF-β, or IL-4 lacked skin sclerosis but developed emphysema, indicating that different genes are involved in the development of skin sclerosis and pulmonary emphysema in TSK/+ mice [17,29]. Furthermore, other studies have shown that the pulmonary pathology remained unchanged in TSK/+ CD4 knockout (CD4-/-) mice [31] and TSK/+ mice treated with an anti-IL-4 antibody [20]. The dermal and pulmonary components of the TSK/+ phenotype can, therefore, be dissociated *in vivo*.

We used a transgenic approach instead of using a recombinant protein for the following reasons:

1. Because the half-life of a recombinant protein is quite short, recombinant protein treatment needs an enormous dose of the active form of HGF protein and frequent injections.

2. Administering a high dose of the active form of the HGF protein could cause adverse effects, such as tumorigenesis in other organs [37].

3. Recombinant protein treatment is very costly.

By contrast, the transgenic approach is simple, safe, and cheap and needs much less frequent injections. Repeated weekly injection of HGF-HVJ liposomes achieves a continuous high plasma level of HGF [14-16].

Although further studies are needed to fully explore the effects of HGF on SSc, it is possible that HGF therapy might be a promising strategy for the treatment of SSc.

## Conclusion

*HGF *gene transfection of TSK/+ mice resulted in a marked reduction of hypodermal thickness, including the subcutaneous connective tissue layer. However, TSK/+-associated defects in pulmonary architecture were unaffected by *HGF *gene transfection. *HGF *gene transfection significantly inhibited the expression of IL-4 and TGF-β1 mRNA in the spleen and skin but not in the lung. Recombinant HGF significantly inhibited IL-4 production by TSK/+ CD4^+ ^T cells stimulated by allogeneic DCs. HGF might represent a novel strategy for the treatment of SSc.

## Abbreviations

CD4-/- = CD4 knockout; DC = dendritic cell; ELISA = enzyme-linked immunosorbent assay; fbn-1 = fibrillin 1; GVHD = graft-versus-host disease; HGF = hepatocyte growth factor; HSCT = hematopoietic stem-cell transplantation; HVJ = hemagglutinating virus of Japan; IFN = interferon; IL = interleukin; IL-4 receptor alpha = IL-4Rα; mAb = monoclonal antibody; MLR = mixed lymphocyte reaction; pa/pa = homozygous pallid; PCR = polymerase chain reaction; RT = reverse transcriptase; SSc = systemic sclerosis; TGF = tumor growth factor; TSK/TSK = homozygous tight skin; TSK/+ = tight skin.

## Competing interests

The authors declare that they have no competing interests.

## Authors' contributions

T Iwasaki conceived the study, participated in the design and co-ordination of the study, and participated in the interpretation of the results. T Imado and SK performed the animal study and histologic analysis. HS participated in the design of the animal study.
